# Supplementary material for: Multi-Approach Characterization of Novel Pyrene-Degrading Mycolicibacterium austroafricanum Isolates Lacking nid Genes
Source: Microorganisms. 2023 May 27;11(6):1413. doi: 10.3390/microorganisms11061413 (PMC10301208; doi:10.3390/microorganisms11061413)
Supplement: Supplementary file 1 [file microorganisms-11-01413-s001.zip › microorganisms-2348677-supplementary.pdf]

Supplementary Figure S1: CG/MS chromatograms of negative controls. (A) BH medium, pyrene and peptone, (B) BH medium, pyrene. Times: T0h and T7 days.

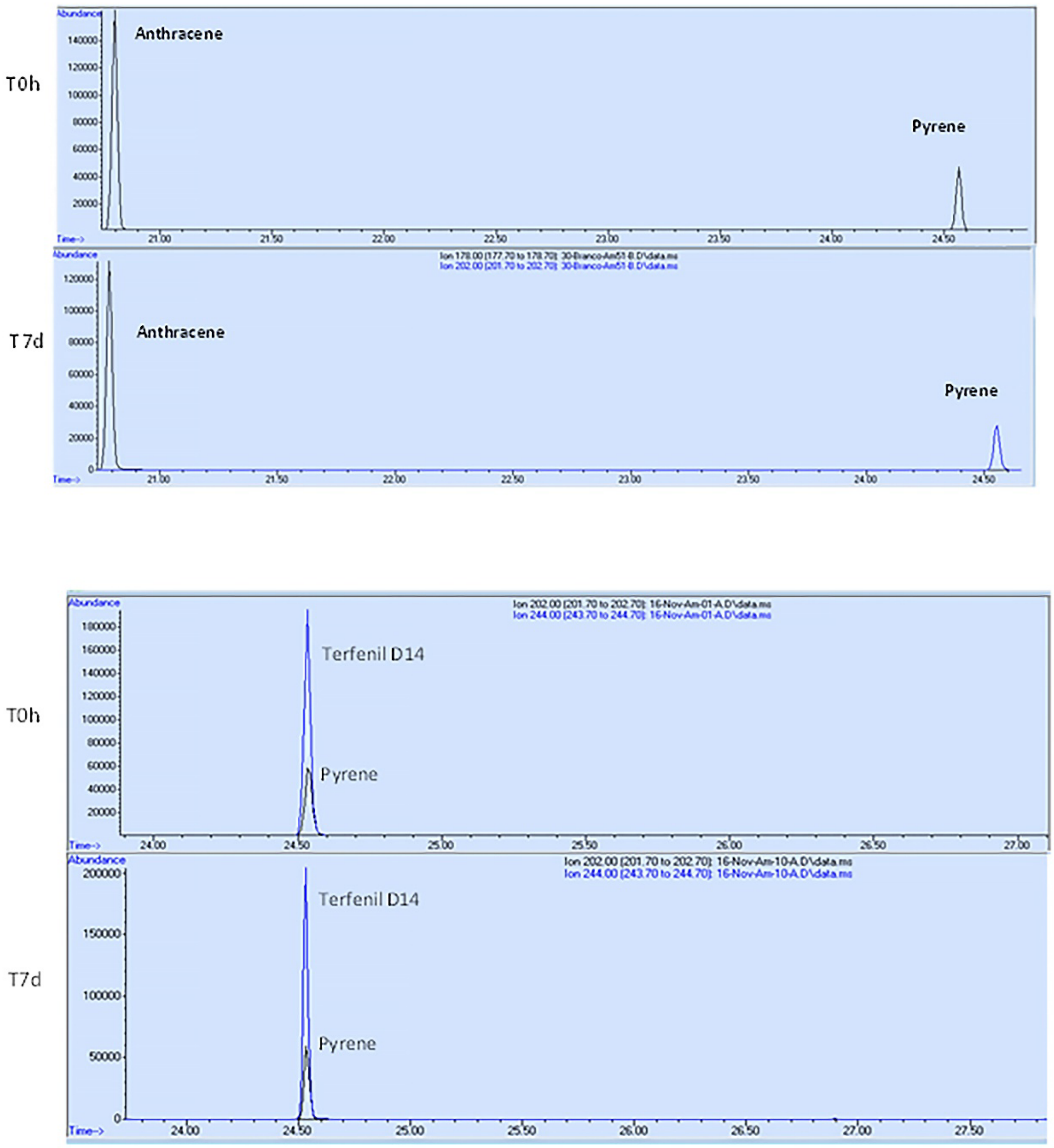

A- Ìon 178 – anthracene (internal standard) and íon 202- pyrene.

B- Ìon 244 – Terfenil – D14 (internal standard) and íon 202- pyrene.

Supplementary Figure S2: CG/MS chromatograms of pyrene biodegradation by MYC038 (A) and MYC040 (B) with internal standard. Times: T0h, T3d and T7days.

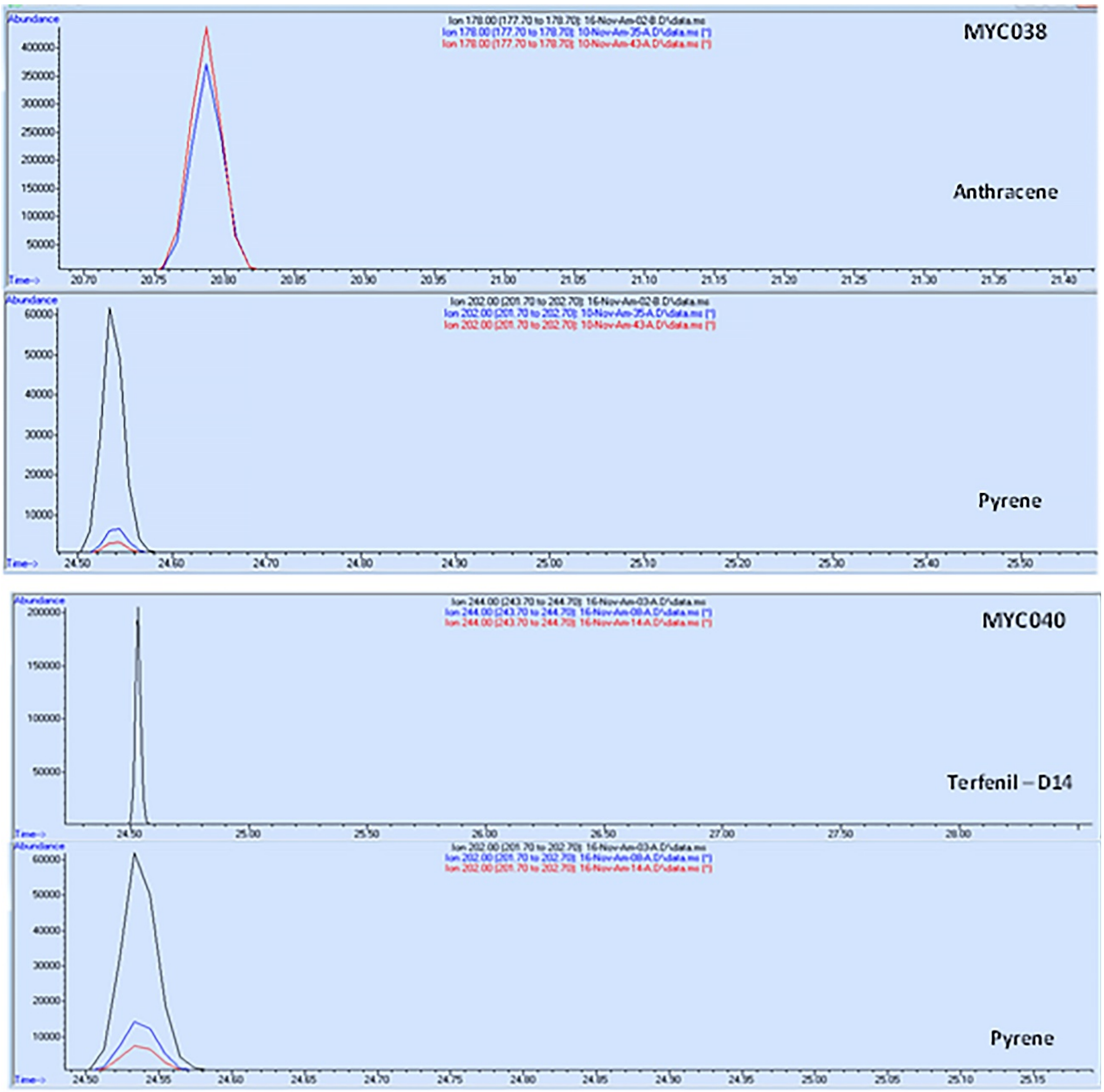

Supplementary Table S1: List of primers used in this study.

Target gene	Primer	Sequence (5'- 3')	Fragment size (bp)	Reference
*nid*A	NidAF	ATGACCACCGAAACAACCGGA	1368	Miller *et al.*, 2004
	NidAR	TCAAGCACGCCCGCCGAATGC		
*nid*A	GPF	CGGCGCCGACAAYTTYGTNGG	262	Cébron *et al.*, 2008
	GPR	GGGGAACACGGTGCCRTGDATRAA		
*nid*A3	nidA3fw	CCTGATGCGACGACAATG	1392	Debruyn *et al.*, 2012
	nidA3rv	TCATACGTTCTCATCCCTTCTC		
*nid*B	NidBF	ATGAACGCGGTTGCGGTCGAT	510	Miller *et al.*, 2004 / This study
	NidBR	CTACAGGACTACCGACAGGTT		
*nid*B3	NidB3F	ACGACAACGGTGCCGCAG	527	This study
	NidB3R	AGGTTGTGCGTGGGCAAC		
*nid*B2	NidB2F	TTGGGTGGGCGCGATCGCGCAC	226	This study
	NidB2R	CAGCAACTCCCACCGGCCGCTG		
*cyp*151	RP1F1	AGCTGGATCCTCAACAAG	209	Brezna *et al.*, 2006
	RP1F2	TCATCGCGATCATGCTC		
*cyp*150	Fm10F1	CCCTACTTCGATCACCTGCGC	1015	Brezna *et al.*, 2008
	Fm10R2	CCGAACGCGATGTGCTCGCG		
